# Suspected Drinking Water Poisoning in a Domestic Kitten with Methemoglobinemia

**DOI:** 10.3390/vetsci8110243

**Published:** 2021-10-20

**Authors:** Francesca Fidanzio, Andrea Corsini, Kevin Pascal Spindler, Serena Crosara

**Affiliations:** Department of Veterinary Sciences, University of Parma, Strada del Taglio, 10, 43126 Parma, Italy; andrea.corsini@unipr.it (A.C.); kevinpascal.spindler@unipr.it (K.P.S.); serena.crosara@unipr.it (S.C.)

**Keywords:** methemoglobinemia, methemoglobin intoxication, drinking water poisoning, kitten, veterinary pediatrics, toxicology

## Abstract

A 4-month-old male indoor cat was referred for dyspnea, mental dullness and weakness, which appeared two days earlier. The cat had been adopted at 3 months of age. Physical exam showed cyanosis, dyspnea and mild hypothermia. The “spot test” was supportive of methemoglobinemia. Co-oximetry blood gas analysis revealed severe methemoglobinemia (81.40%), severe hyperchloremia and mild hyponatremia. CBC, biochemistry and urinalysis were within normal limits, blood smear showed the presence of Heinz bodies. Treatment was instituted with oxygen therapy, methylene blue 1% solution, ascorbic acid, intravenous fluid therapy. The clinical course was favorable with rapid improvement of cyanosis and methemoglobinemia (4.2%). Acquired methemoglobinemia was hypothesized. Two weeks after discharge the cat was asymptomatic but mild methemoglobinemia (15.60%) and hyperchloremia were evident. Exposure to oxidants contained in drinking water was suspected so the owners were instructed to use bottled water only. One month later the cat was asymptomatic, and methemoglobinemia and chloremia were within normal limits. Even if a congenital form due to cytochrome b5 reductase deficiency cannot be ruled out, drinking water intoxication is the most likely cause of methemoglobinemia in this cat.

## 1. Introduction

Methemoglobin (MetHb) results from oxidation of the iron in hemoglobin from ferrous iron (Fe^2+^) to ferric iron (Fe^3+^). Methemoglobin cannot carry oxygen [1,2]. MetHb accumulation may occur due to an excess of oxidizing molecules saturating protective mechanisms (acquired methemoglobinemia) [3,4] or inability to reduce MetHb, mainly due to genetic abnormalities (congenital methemoglobinemia) [2,5,6].

This report describes a case of toxic methemoglobinemia in a 4-month-old cat, induced by suspected exposure to oxidizing substances contained in drinking water.

## 2. Case Presentation

A 4-month-old domestic shorthair tomcat, weighing 2 kg, was referred to the Veterinary Teaching Hospital of the University of Parma for dyspnea, mental dullness, decreased appetite and weakness in the last two days. The cat had been adopted at approximately 3 months of age and always lived indoor; the owners did not report any symptoms at the time of adoption. The kitten was regularly vaccinated and fed with a dry commercial feline diet. Access to toxic substances could not be excluded because bottles of chlorinated detergents were present in the room where the cat litter box was located; however, bottles were closed and any contact with these substances was deemed unlikely by the owners.

**Figure 1:**
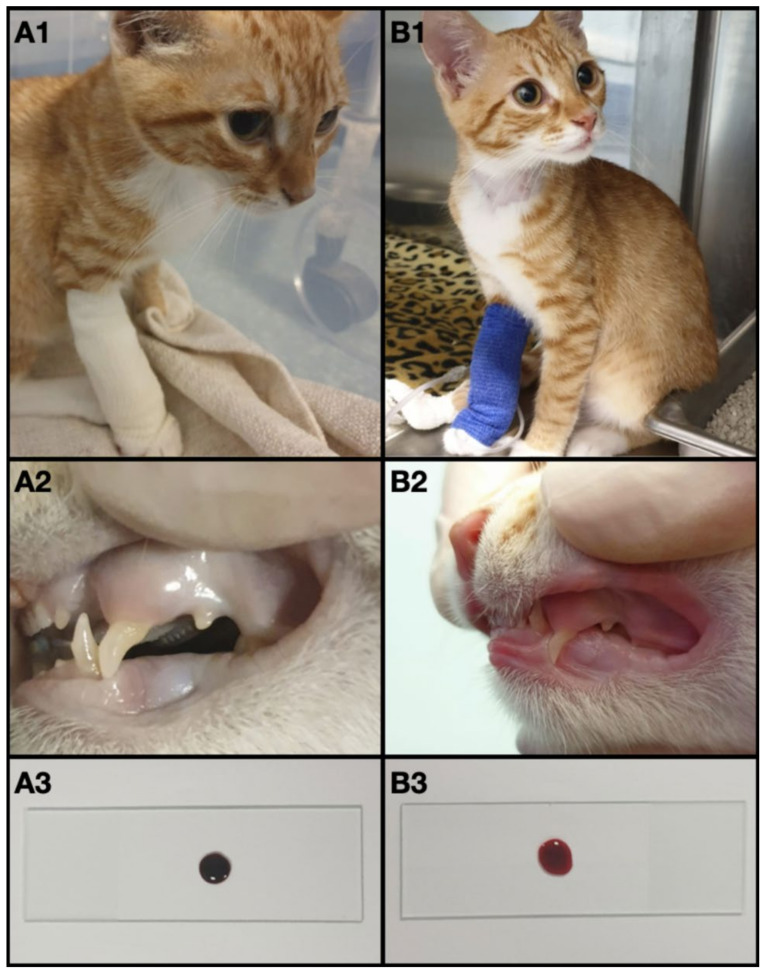
(**A1**) the kitten at admission. (**A2**) Cyanotic oral mucous membranes at admission. (**A3**) blood drop at admission. (**B1**) the kitten three hours after methylene blue administration. (**B2**) pink oral mucous membranes three hours after methylene blue administration. (**B3**) blood drop three hours after methylene blue administration.

Physical exam showed cyanotic oral and preputial mucous membranes and foot pads (Figure 1(A1,A2)). The cat was mildly hypothermic (36.9 °C), the heart rate was 160 bpm and the systolic blood pressure, measured with Doppler, was 125 mmHg. Cardiac and pulmonary auscultation were unremarkable, no cardiac murmur was audible, femoral pulses were concordant. Thoracic radiographs performed in latero-later and dorsoventral views showed no abnormalities.

**Figure 2:**
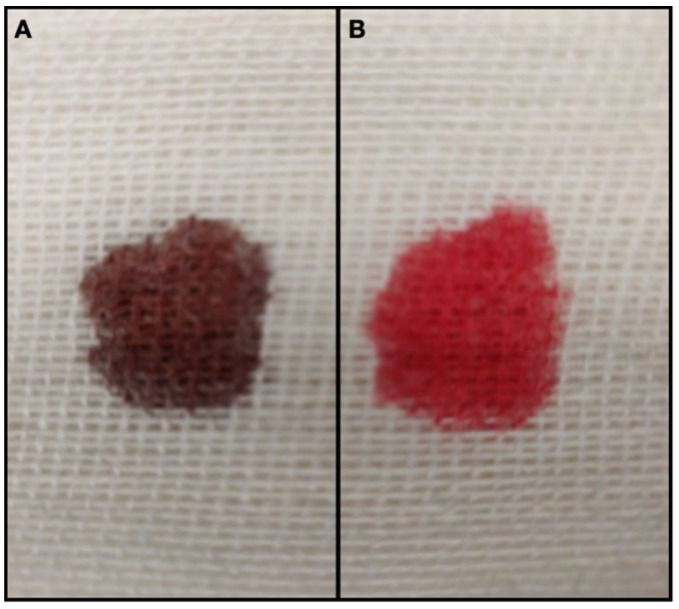
Methemoglobine spot test. (**A**) At admission the blood remains noticeably brown in color (MetHb 81.4%). (**B**) Three hours after the administration of methylene blue the blood was bright red (Met 4.2%).

A blood sample was collected from the jugular vein to run a minimum database. As the sample was collected, the blood appeared chocolate brown-colored (Figure 1(A3)). A “methemoglobin spot test” was performed placing a drop of fresh blood on a white absorbent material (gauze) and comparing it to a color chart (Figure 2), as previously described [7].

Methemoglobinemia was suspected and confirmed by venous blood gas analysis with co-oximetry (ABL 800 Flex, Radiometer) which revealed severe methemoglobinemia (81.40%; reference range 0–1%), metabolic acidosis with severe hyperchloremia (321 mmol/L; reference range 105–116 mmol/L) and mild hyponatremia (142 mmol/L; reference range 151–158 mmol/L) (Table 1).

**Figure 3:**
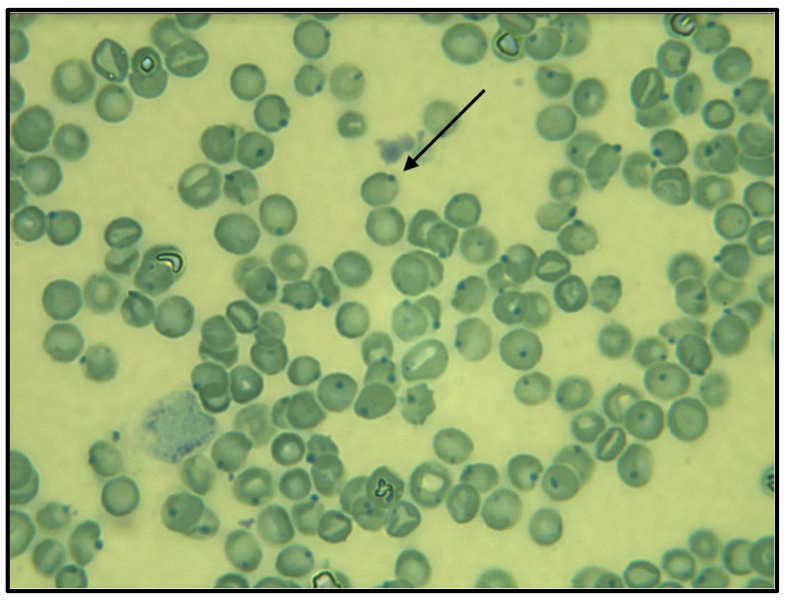
Heinz bodies (arrow) in the blood smear. Blu cresile stain, ×100 objective lens.

CBC, serum chemistry, and urinalysis were unremarkable (Table 2); the blood smear revealed several Heinz bodies, suggestive of oxidation (Figure 3).

The cat was admitted to the intensive care unit and was treated with oxygen therapy (oxygen cage, inspired oxygen concentration 40%) and intravenous fluid therapy with Ringer lactate CRI 2 mL/kg/h. However, despite the oxygen supplementation, dyspnea and cyanosis persisted. The methemoglobinemia was treated with methylene blue 1% solution 1 mg/kg IV over 5 min [2,8] and ascorbic acid 30 mg/kg every 6 h. Cyanosis resolved within a few minutes after methylene blue was administered, mental status improved, and MetHb decrease (4.2%; reference range 0–1%) was highlighted in a few hours (Figure 1(B1–B3) and Figure 2); hyperchloremia (254 mmol/L; reference range 105–116 mmol/L) was persistent (Table 1). Serial venous blood gas analyses with co-oximetry were performed during hospitalization. Mild hyperchloremia (132 mmol/L; reference range 105–116 mmol/L) and mild methemoglobinemia (3.7%; reference range 0–1%) were detected on discharge after 48 h (Table 1).

The diagnostic suspect was acquired methemoglobinemia. The cat was discharged with the indication to avoid stored foods that could contain nitrites/nitrates (e.g., dried meat, canned meat, marinated fish) and to prevent access to toxic substances or drugs (e.g., chlorinated detergents, acetaminophen). It was also indicated to feed the cat exclusively with a commercial kitten dry food.

Two weeks later, during a scheduled recheck, the owners reported that they had carefully followed the advice and that the cat was bright and alert with normal appetite. The physical exam was unremarkable; mucous membranes were pink. Venous blood gas analysis and serum chemistry were performed and moderate methemoglobinemia (15.60%; reference range 0–1%) with hyperchloremia (409 mmol/L; reference range 105–116 mmol/L) were evident. The cat was hospitalized but it was decided to not administer methylene blue due to concerns for potential oxidative damage following repeated dosing, and the cat was treated with intravenous fluid therapy (Ringer lactate).

A possible poisoning by chlorinated drinking water was hypothesized. The cat was discharged with the indication to use only bottled water, for the suspected exposure to oxidizing substances contained in tap water.

One month later the cat was asymptomatic and both MetHb (1.5%; reference range 0–1%) and chloride (109 mmol/L; reference range 105–116 mmol/L) were within the reference ranges. The owner reported no symptoms to subsequent follow-up phone calls at three and six months.

## 3. Discussion

Methemoglobinemia has been reported due to congenital etiology or secondary to intoxication. Congenital methemoglobinemia in dogs and cats, like in humans, is mostly caused by the deficiency of cytochrome b_5_ reductase (CYB5R), which is responsible of hemoglobin reduction [2,5]. The acquired form can be caused by oxidant chemicals and drugs that are capable of inducing methemoglobinemia [9]. Furthermore, cats cannot readily metabolize and conjugate certain drugs (i.e., acetaminophen) [5].

In this kitten, the simultaneous increase in chloremia and MetHb at the blood gas with co-oximetry, along with the presence of Heinz bodies at the blood smear, suggests as a first hypothesis oxidative damage secondary to intoxication. In course of congenital methemoglobinemia the presence of hyperchloremia is not expected; moreover, the presence of Heinz bodies is described in both congenital and acquired forms, but it is strongly suggestive of the latest.

The accurate medical history excluded exposure to drugs (i.e., acetaminophen, sulphonamides, phenazopyridine, local anesthetics) [10,11,12] or toxic (i.e., chlorinated detergents, chlorinated pool water) [13] that can cause methemoglobinemia. Moreover, the cat did not present any alteration of liver enzymes activity that are usually elevated in course of acetaminophen intoxication. The exposure to nitrites/nitrates, used for the storage of certain preserved foods, has been described as possible cause of methemoglobinemia in humans [14]. The presence of these substances in the cat’s food was not excluded by mean of chemical analysis at the first presentation. However, the cat was exclusively fed with a different commercial feline diet since the first discharge and the resolution of methemoglobinemia was obtained only after the drinking water was changed. Thus, food contamination as the cause of the intoxication seems extremely unlikely.

At presentation, the cat presented a severe hyperchloremia associated with the methemoglobinemia; therefore, we explored other possible causes of hyperchloremia, such as potassium bromide or spironolactone or acetazolamide therapy, diarrhea, iatrogenic, salt intoxication, diabetes mellitus, renal failure, strenuous exercise [15]. All these causes were ruled out based on the medical history and blood and urine analysis results. Moreover, the hyperchloremia at presentation (321 mmol/L), higher than natremia (142 mmol/L), was not compatible with life and with the principle of electroneutrality. The manual of the blood gas analytic instrument reported possible interference in the measurement of chloride in the presence of halogens in the sample (e.g., Br-, I-, ClO4-) [16]. Substances such as potassium bromate, chlorates, nitrites/nitrates may be present in drinking water after purification processes [17]; indeed, few cases of acquired methemoglobinemia in children and pregnant women are reported because of chronic drinking water intake [18,19]. Based on these findings, we hypothesized a possible intoxication by tap water and we recommended using bottled water. Physical chemical analysis to assess the presence of bromate, chlorates, nitrites/nitrates in the tap water would have given us more information, but it was not performed because we could not find a certified laboratory for the research of these substances in the tap water. However, after one month of exclusive use of bottled water, the cat had normal MetHb and chloremia, further supporting our hypothesis. The evaluation of CYB5R activity was not performed. It would have been important to definitively exclude congenital methemoglobinemia. Despite this, the acute onset of symptoms and the resolution of methemoglobinemia at the follow-up make a congenital form due to CYB5R deficiency unlikely.

## 4. Conclusions

Our report describes possible tap water intoxication in a kitten with methemoglobinemia and hyperchloremia. The treatment with methylene blue was safe and effective for the resolution of cyanosis. The indication to feed commercial diet and bottled water led to complete resolution of methemoglobinemia at long-term follow-up.

## Figures and Tables

**Table 1:** Serial blood gas analyses prior and after administration of methylene blue.

Day	1	1	2	3	Reference Interval
Time	07:02 p.m.	11:57 p.m. *	07.37 a.m.	07:25 a.m.	
pH	7.249	7.332	7.343	7.418	7.26–7.46
pCO2	40.2	30	35.5	25.5	32.7–42.7 mmHg
HCO3	16.9	15.7	18.7	18.3	18–23.5 mmol/L
Hematocrit	40.9	40.8	41.8	39.5	39–54%
MetHb	81.4	4.2	2.9	3.7	0–1%
Sodium	142	147	151	151	151–158 mmol/L
Chloride	321	254	216	132	105–116 mmol/L

* 3 h post intravenous methylene blue administration pCO2 = partial pressure of carbon dioxide HCO3 = bicarbonate.

**Table 2:** Selected clinicopathological parameters at the admission.

Laboratory Variables	Results	Reference Interval
AST	15	14–41 U/l
ALT	17	5–45 U/l
CK	196	91–326 U/l
ALP	46	0–120 U/l
Creatinine	0.62	0.8–1.80 mg/dl
Urea	43.30	15–60 mg/dl
Glucose	108	75–160 mg/dl
Total proteins	6.55	6.0–8.0 g/dl
Albumin	2.87	2.10–3.30 g/dl
Hematocrit	40.1	24–45%
Platelets	400,000	300,000–700,000/mm^3^
Leucocytes	7340	5000–19,000/mm^3^
USG	1036	>1032

AST = aspartate aminotransferase; ALT = alanine aminotransferase; CK = creatine kinase; ALP = Alkaline Phosphatase; USG = urine specific gravity.
